# Fragility and Tendency to Crystallization for Structurally Related Compounds

**DOI:** 10.3390/ijms25063200

**Published:** 2024-03-11

**Authors:** Katarzyna Grzybowska, Zaneta Wojnarowska, Andrzej Grzybowski, Marian Paluch

**Affiliations:** Institute of Physics, University of Silesia in Katowice, ul. 75 Pulku Piechoty 1, 41-500 Chorzów, Poland

**Keywords:** amorphous materials, glass transition, supercooled liquid, physical stability, crystallization, molecular dynamics, fragility, thermodynamic fragility

## Abstract

The present study was designed to investigate the physical stability of three organic materials with similar chemical structures. The examined compounds revealed completely different crystallization tendencies in their supercooled liquid states and were classified into three distinct classes based on their tendency to crystallize. (S)-4-Benzyl-2-oxazolidinone easily crystallizes during cooling from the melt; (S)-4-Benzylthiazolidine-2-thione does not crystallize during cooling from the melt, but crystallizes easily during subsequent reheating above *T*_g_; and (S)-4-Benzyloxazolidine-2-thione does not crystallize either during cooling from the melt or during reheating. Such different tendencies to crystallize are observed despite the very similar chemical structures of the compounds, which only differ in oxide or sulfur atoms in one of their rings. We also studied the isothermal crystallization kinetics of the materials that were shown to transform into a crystalline state. Molecular dynamics and thermal properties were thoroughly investigated using broadband dielectric spectroscopy, as well as conventional and temperature-modulated differential scanning calorimetry in the wide temperature range. It was found that all three glass formers have the same dynamic fragility (*m* = 93), calculated directly from dielectric structural relaxation times. This result verifies that dynamic fragility is not related to the tendency to crystallize. In addition, thermodynamic fragility predictions were also made using calorimetric data. It was found that the thermodynamic fragility evaluated based on the width of the glass transition, observed in the temperature dependence of heat capacity, correlates best with the tendency to crystallize.

## 1. Introduction

Amorphous states do not exhibit the long-range order found in crystalline solids. The structural disorder in amorphous systems imparts distinctive properties that make them suitable for various industrial, pharmaceutical, and biological uses. Many important materials, such as polymers, ceramics, metals, optical materials (glasses and fibers), foods, and pharmaceuticals exist in a disordered amorphous solid state [1,2]. Among these materials, the amorphous state is especially relevant for formulating pharmaceuticals. This usually stems from the higher solubility and higher dissolution rates of amorphous drugs as compared to their crystalline counterparts. It has been established that the solubility and dissolution rate in water of many amorphous pharmaceuticals are orders of magnitude greater than their crystalline counterparts [3,4,5,6,7,8,9,10]. Unfortunately, the amorphous state is commonly physically unstable because its greater molecular mobility relative to the crystalline state often lowers the activation barriers for the crystallization process. As a result, amorphous materials can return to their thermodynamically stable crystalline forms during manufacturing, storage, or dissolution [11,12,13,14,15]. Therefore, it is crucial to understand how molecular mobility changes near the glass transition and, consequently, how it affects the physical stability of glass-forming liquids.

The liquid-glass transition is related to the structural α-relaxation, which is the dominant relaxation in the supercooled liquid state. This relaxation reflects the cooperative and correlated motions of many molecules together. In most cases, glass-forming liquids exhibit a dramatic slowing down of the α-relaxation upon cooling toward the glass transition temperature *T*_g_. Such a peculiar temperature dependence of the structural α-relaxation times *τ*_α_(*T*) of supercooled liquids cannot be described by a basic Arrhenius tendency, and it is one of the important topics of current condensed matter physics [16,17,18,19,20,21,22]. The degree of deviation of the dependence *τ*_α_(*T*) from Arrhenius law in the vicinity of *T*_g_ provides significant information to characterize glass-forming liquids, and it can be described by the fragility parameter *m* (or steepness index), which is a derivative measure of the deviation from the Arrhenius dependence near *T*_g_. The fragility parameter *m* has been popularized by Angell and many other authors [23,24,25] as a measure to characterize the sensitivity of the molecular mobility of supercooled liquids to temperature changes near the glass transition. According to the concept, glass-forming liquids are classified between two extremes: “strong” glass formers showing Arrhenius behavior in the temperature dependence of log_10_(*τ_α_*) in the Angell plot, and “fragile” glass formers that deviate significantly from the Arrhenius equation. In other words, the molecular mobility of “fragile” liquids varies rapidly upon approaching the glass transition in contrast to that of “strong” materials.

In recent years, many studies have been carried out in search of a correlation between the fragility parameter and the tendency of materials to crystallize from supercooled liquid and glassy states, as well as the ability of supercooled liquids to form glasses [10,26,27]. Consequently, the fragility parameter *m* has often been used to predict the physical stability and the glass-forming ability of amorphous pharmaceuticals and other glass formers. Some recent theoretical considerations [28,29] have argued that the fragility index should be calculated based on temperature diffusivity dependencies instead of those of the structural relaxation time *τ_α_* or viscosity *η*, especially when investigating a correlation between the dynamic fragility parameter and the nucleation and crystal growth in the context of the classical nucleation theory. Nevertheless, the diffusion coefficient measurements near *T*_g_ are rarely performed, whereas evaluations of the diffusion coefficient from *τ_α_* or *η* based on well-known decoupling relations can be inaccurate. Thus, for many decades, independently of the experimental technique used to determine the dynamic fragility, it has been believed that the physical stability of amorphous systems is better if they are stronger materials, i.e., with smaller values of the fragility parameter [11,30,31,32,33,34,35,36,37].

However, recent experimental studies devoted to amorphous materials have indicated some exceptions regarding the suggested correlation between fragility and tendency to crystallize for pure drugs, as well as pharmaceutical compositions [10,38,39,40,41,42,43,44]. Lately, some interesting reports have been published [45,46,47] on crystallization kinetics and inter-conformer relaxation dynamics, invoking discussion about the dynamic fragility parameter that does also not reflect the crystallization properties of materials characterized by similar chemical structures. The results suggest that a single fragility parameter *m* may not be sufficient to predict the diverse crystallization mechanisms observed for various glass formers characterized by different properties, such as various chemical structures, molecular interactions (hydrogen bonding or ionic interactions, ability to form special intermolecular structures like dimers, etc.), flexibility and conformational changes, or chemical reactions (i.e., ability to tautomerize). 

In this paper, we investigated isobaric dynamic fragility *m* as well as a thermodynamic measure of steepness index for three small molecular weight organic compounds: (S)-4-Benzyloxazolidine-2-thione, (S)-4-Benzyl-2-oxazolidinone, and (S)-4-Benzylthiazolidine-2-thione, which have remarkably similar molecular structures. Despite their similar structures, we found that they have completely different crystallization tendencies. By investigating molecular dynamics using broadband dielectric spectroscopy (BDS) as well as thermal properties using differential scanning calorimetry (DSC), we verified whether fragility parameters can be used to predict their physical stability. Our work also covered the study of crystallization kinetics in relation to the dynamic and thermodynamic fragility measures. Based on performed analyses, one can suggest that there is a feature of the glass transition, measurable in the DSC experiment, which may be useful in predicting the crystallization tendency of compounds. 

## 2. Results and Discussion

### 2.1. Calorimetric Study of Tendency to Crystallize

**Figure 1:**
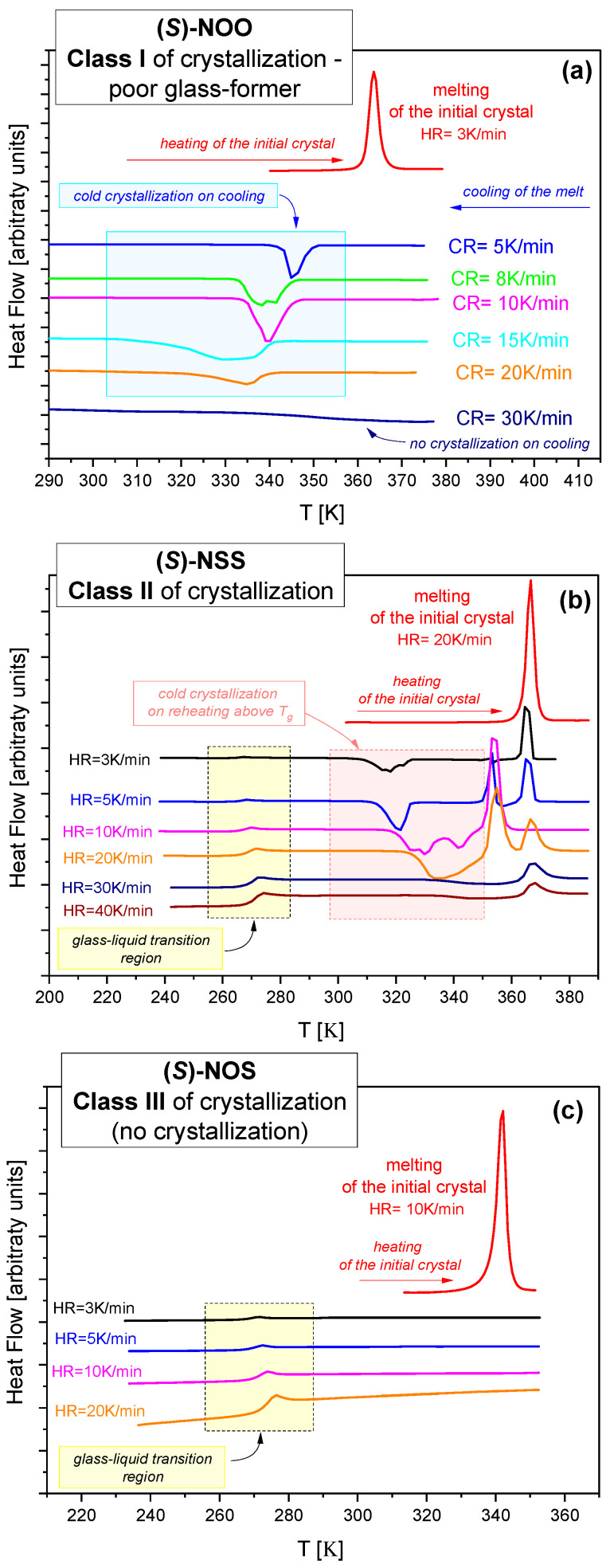
DSC thermograms of the examined materials measured at different cooling and heating rates obtained for (**a**) (S)-4-Benzyl-2-oxazolidinone ((S)-NOO), (**b**) (S)-4-Benzylthiazolidine-2-thione ((S)-NSS), and (**c**) (S)-4-Benzyloxazolidine-2-thione ((S)-NOS).

To thoroughly recognize the tendency to recrystallization of the examined materials in their supercooled liquid states, we subjected each melt sample to multiple cooling and heating cycles in a wide temperature range (i.e., below their glass transition temperatures and above melting points) at different rates (from 3 to 40 K/min). However, for a single measurement cycle (i.e., cooling and heating), the cooling rate was always equal to the heating rate (CR = HR). The obtained DSC thermograms for examined compounds are presented in Figure 1a–c. 

It was found that (S)-4-Benzyl-2-oxazolidinone [(S)-NOO)] is a poor glass former (see Figure 1a). It was difficult to supercool its melt to obtain a glassy state. The compound easily crystallized while cooling from the melt, which was observed on thermograms as exothermic thermal effects. Only relatively rapid cooling (CR ≥ 30 K/min) allowed the glassy state to be achieved.

A different tendency to crystallize was observed in (S)-4-Benzylthiazolidine-2-thione [(S)-NSS)] (see Figure 1b). For this compound, no crystallization was observed when cooling from the melt to below *T_g_* (not shown in Figure 1), even if the cooling rate was very slow (3 K/min). However, this good glass-former easily crystallized when it was reheated to above the glass transition temperature. Exothermic effects were observed even for relatively fast heating rates (HR = 40 K/min). The cold crystallization processes were complicated into different polymorphic forms. 

In the case of (S)-4-Benzyloxazolidine-2-thione ((S)-NOS), no crystallization was observed, either during the quenching from the melt (not shown) or even during very slow heating (3 K/min) above T_g_ in the supercooled liquid state *Figure 1c). High resistance to crystallization indicates that this compound is a very good glass-forming material, characterized by high stability.

After analyzing the thermograms presented in Figure 1a–c, one can conclude that the investigated materials have completely different tendencies to crystallize, although they are very similar in chemical structure. We classified them into three different “classes of crystallization”, which were originally proposed by Baird et al. [27] and then widely used by many research groups, mainly in studies of the physical stability of drugs [48,49,50,51]. The detailed methodology for testing materials to discover which class they belong to is described in [27], including the cooling/heating rates that typically cover 5–20 K/min. 

According to this classification, our materials can be classified as follows:

- Class I. For (S)-NOO, crystallization was observed during cooling from the melt (see Figure 1a). It is a poor glass former that requires exceeding a critical cooling rate (~30 K/min) for the successful formation of glass.
- Class II. (S)-NSS did not crystallize during cooling from the melt to below *T_g_*, but easily crystallized during subsequent reheating above *T*_g_ (see Figure 1b).
- Class III. For (S)-NOS, no crystallization was observed, either during the quenching from the melt or during the reheating cycle (see Figure 1c). This compound is characterized by the best physical stability among those tested.

### 2.2. Molecular Dynamics Investigations (Dielectric Study)

**Figure 2:**
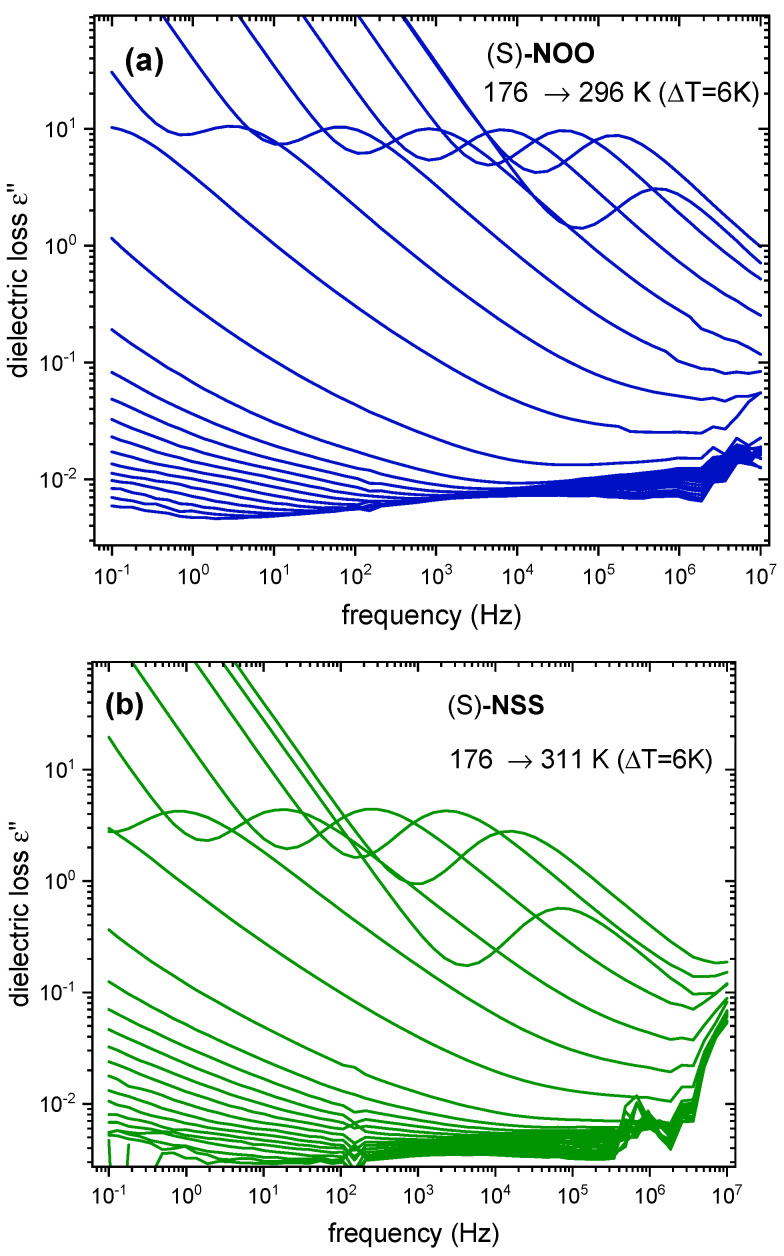

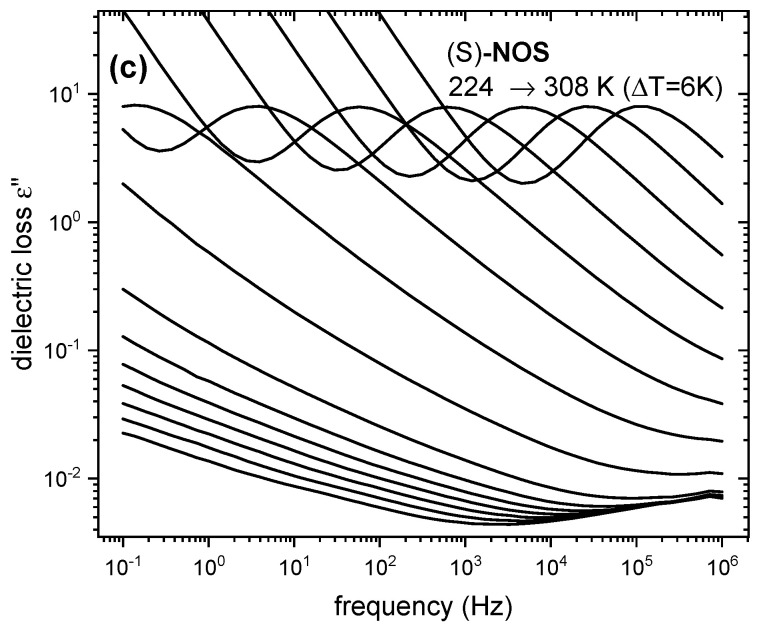
Results of the BDS measurements for the examined materials in the supercooled liquid state obtained during heating from the glassy state: (**a**) dielectric spectra for (S)-NOO, (**b**) for (S)-NSS, and (**c**) for (S)-NOS.

To find a potential correlation between molecular mobility and the tendency to crystallize, we performed molecular dynamics investigations of examined materials. For this purpose, we applied broadband dielectric spectroscopy measurements because BDS is an efficient technique for studying molecular mobility over many decades of timescale and at different temperatures and pressures. The selected dielectric spectra for (S)-NOO, (S)-NSS, and (c) (S)-NOS obtained in the wide temperature range during the heating of the samples are presented in Figure 2. As can be seen, all samples in the supercooled liquid state revealed well-separated α-relaxation, which is the dominant relaxation process at temperatures *T* > *T_g_*. This relaxation is related to the liquid-glass transition and reflects the cooperative and correlated motions of many molecules together. It is often believed that this global relaxation may have a key impact on recrystallization from a supercooled liquid and glassy state. As can be observed in Figure 2, when materials are heated, the α-relaxation peak shifts to higher frequencies for all examined compounds. This indicates that their global molecular mobility increases. By analyzing structural relaxation in dielectric spectra, we can also identify the beginning of the cold crystallization process. As can be seen in Figure 2a,b for (S)-NOO and (S)-NSS, respectively, the amplitude of the α-process (i.e., the dielectric strength Δ*ε*_α_), proportional to the total amount of relaxing units participating in the structural relaxation, began to decrease at given temperatures on heating of samples. The decline in Δ*ε*_α_ (T) was attributed to the initiation of cold crystallization and indicated the increasing degree of crystallinity in the samples. For the non-crystallizing (S)-NOS, we did not observe such a phenomenon of Δ*ε*_α_ drop, and the magnitude of the α-relaxation peaks for this compound were almost constant during heating. 

To evaluate the relaxation times of α-relaxation at various temperatures for the examined materials, we fitted the entire dielectric spectra using the following Havriliak-Negami (HN) formula [52,53]:

(1) $$\varepsilon *\left(\omega \right)=\varepsilon '\left(\omega \right)-i\varepsilon ''\left(\omega \right)=\varepsilon_{\infty }+\sum_{k}\frac{\Delta \varepsilon_{k}}{{\left[1+{\left(i\omega \tau_{k}\right)}^{\xi_{k}}\right]}^{\delta_{k}}},$$

where *ε*_∞_ is the high-frequency limit permittivity, *k* stands for either the primary and the secondary processes, Δ*ε*_k_ is the relaxation strength, *τ*_k_ is the HN relaxation time, and *ξ*_k_ and *δ*_k_ are the HN exponents of the relaxation processes. 

**Figure 3:**
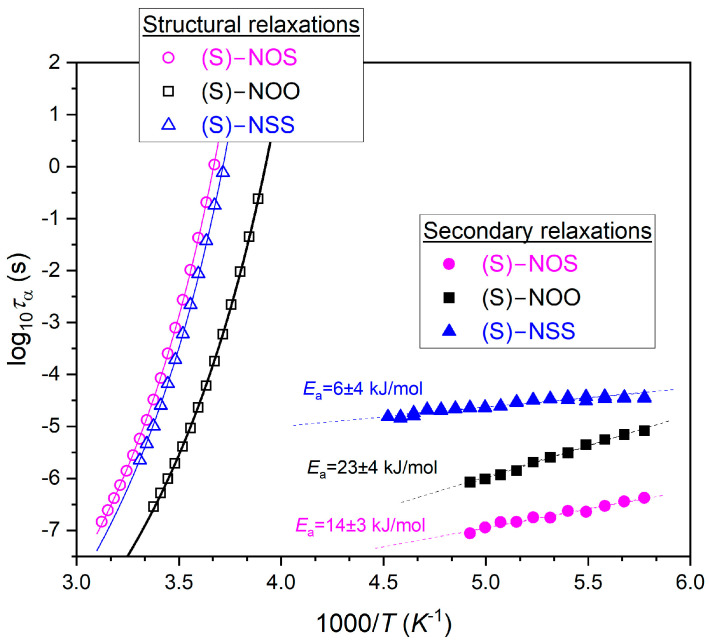
Temperature dependencies of structural and secondary relaxation times for (S)-NOS (magenta circles), (S)-NOO (black squares), and for (S)-NSS (blue triangles), as well as their fits to the VFT equation given by Equation (3) (structural relaxations), and to the Arrhenius law (secondary relaxations).

From the best fits of dielectric spectra, as shown in Figure 2, we found the temperature dependencies of structural (α) and secondary relaxation times (see Figure 3). Each material revealed only one relatively well distinguished secondary relaxation of a small magnitude and small activation energy, shown in Figure 3. This suggests an intramolecular origin for the secondary relaxations. Based on the dependencies on *τ*_α_(*T*), we can determine the dynamic fragility *m*. It should be noted that the parameter *m* can be defined in various ways, but the dynamic definition [24] given below is widely accepted as the most accurate representation of the physical meaning of the steepness index:

(2) $$m_{P}={\left.\frac{dlog_{10}\tau_{\alpha }}{d\left(T_{g}/T\right)}\right|}_{T=T_{g}}$$

To describe the obtained temperature dependence of the structural α-relaxation times *τ*_α_, we used the Vogel-Fulcher-Tammann (VFT) equation [54,55,56]:

(3) $$\tau_{\alpha }\left(T\right)=\tau_{0}\exp\left(\frac{A}{T-T_{0}}\right),$$

where *τ*_0_, *T*_0_, and *A* are fitting parameters. VFT expression is sometimes adapted by substituting the parameter *A* with the expression *DT*_0_, where *D* is the strength parameter (related to the fragility). From the fits of the dielectric *τ*_α_(*T*) dependencies to the VFT equation, we determined the dielectric glass transition temperatures *T*_g_ of the examined systems. Herein, we have applied the most frequently used definition, according to which *T*_g_ is the temperature at *τ*_α_ = 100 s. The values of the VFT parameters (*τ*_0_, *T*_0_, *A*), as well as values of *T*_g_ for the investigated materials, are collected in Table 1.

On the basis of the VFT fit parameters presented in Figure 3, we calculated values of the dynamic fragility *m* according to Equation (2). It should be noted that in terms of the fragility parameter values, glass-forming liquids can be classified as “strong”, “moderately fragile”, or “fragile” materials, usually using the following ranges of the value of steepness index: *m* ≤ 30, 30 < *m* < 100, and *m* ≥ 100, respectively. Considering the strength parameter *D* in the VFT equation, fragile systems exhibit small values of *D* (*D* < 10), while strong materials possess large values (*D* > 10). Moreover, the fragility parameter *m* is closely related to the activation energy for α-relaxation *E_a_* at *T*_g_, *E_a_*(*T_g_*) = *mRT*ln(10), where the isobaric activation energy is defined [57] as *E_a_*(*T*) = *Rd*ln*τ*_α_/*d*(1/*T*) at a constant pressure, and *R* is the gas constant. This means that fragile liquids are characterized by a higher ratio *E_a_*(*T_g_*)/*T_g_* in comparison with strong materials [10]. Values of dynamic fragility obtained from Equation (2), the strength parameter *D* (related to the fragility), as well as for α-relaxation *E_a_* at *T*_g_, are collected in Table 2. 

We have established that all examined compounds, although they differ completely in their tendency to crystallize, have the same values of dynamic fragility (*m* = 93). Additionally, related to the fragility, they all possess a strength parameter of *D* ≈ 8.5, evaluated from the VFT equation. The obtained fragility values allow the tested substances to be classified as moderately fragile glass-formers, which indicates that they exhibit a similar and significant molecular mobility near *T_g_*.

**Figure 4:**
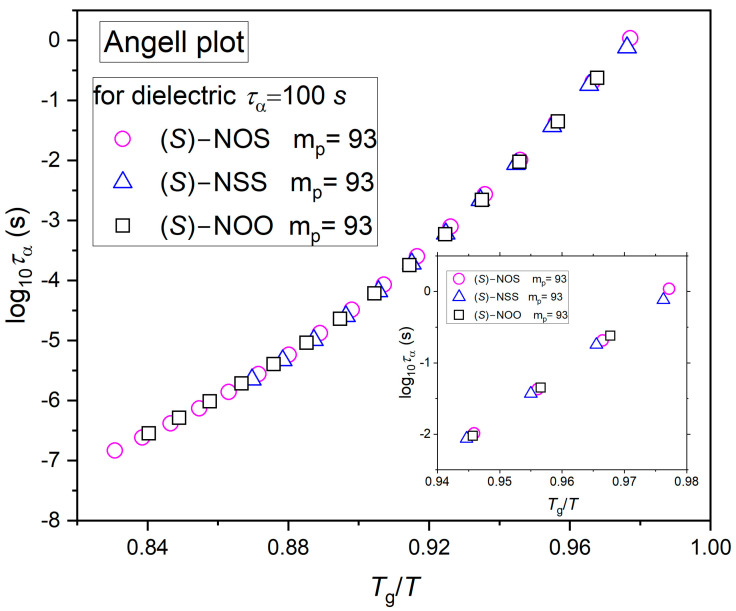
Angell’s plot of the structural relaxation times as a function of normalized temperature *T*_g_/*T* for all three investigated compounds. The inset shows a zoom in the region of the high dielectric relaxation times.

The steepness index *m* of different materials can be graphically represented using the Angell plot [17] using either experimental structural relaxation times or viscosity data. In this plot, the logarithm of the α-relaxation times log_10_(*τ_α_*) or viscosity log_10_(*η*) is plotted versus *T*_g_/*T*. *T*_g_ is usually defined as the temperature at which the structural relaxation time equals 100 s, or at which the viscosity reaches 10^12^ Pa s. As a result, all curves meet at the same point at *T*_g_/*T* = 1. Based on the Angell plot, the value of *m* is measured as the slope of log_10_(*τ_α_*) or log_10_(*η*) plotted versus *T_g_*/*T*, evaluated at *T_g_*. To verify the obtained equality of the fragility values for all materials, we have also prepared the so-called Angell plot, which is only based on experimentally determined structural relaxation times as a function of *T*_g_/*T*, independent of any fitting model (see Figure 4).

As can be seen in Figure 4, the same dynamic fragility values are confirmed by the Angell plot, in which the dependencies of α-relaxation times as a function of *T*_g_/*T* superimpose for all the tested compounds. This means that the examined compounds exhibit the same sensitivity as global molecular mobility to changes in temperature. Therefore, based on the considered prediction, our substances should have rather poor physical stability and a similar tendency to crystallize. However, we obtained the same values of *m* for all compounds despite the fact that they were characterized by completely different degrees of physical stability, which enabled their classification into classes I, II, and III of crystallization tendency. This is an important result because this is another example of the idea that dynamic fragility derived from dielectric measurements of the global molecular dynamics in terms of Equation (2) does not always correctly predict physical stability, even for materials that have remarkably similar molecular structures [10,43,44]. 

**Figure 5:**
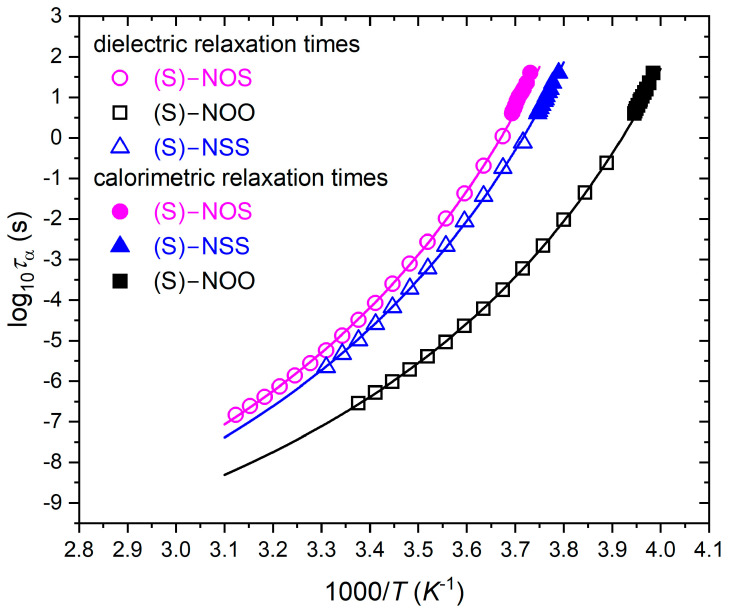
The temperature dependencies of structural relaxation times, determined on the basis of the dielectric (open symbols) and calorimetric (solid symbols) measurements.

Since dielectric spectroscopy can measure relaxation times from only the rotational motion of molecules having permanent dipole moments, it is reasonable to compare them with relaxation times determined from calorimetric measurements, which take into account all types of molecular motions (rotations, translations, vibrations). Therefore, in the next step of our analysis, we checked whether the molecular dynamics from dielectric measurements are related to the molecular mobility determined from calorimetric measurements. To determine structural relaxation times, we performed a stochastic temperature modulated DSC (TOPEM^®^). From the temperature dependencies of the complex heat capacity of the investigated materials, we determined calorimetric relaxation times *τ*_α_ = 1/(2π*f*_max_) at temperatures near *T*_g_ for different frequencies in the glass transition region (the method for determining calorimetric relaxation times is described in Section 3). A comparison of the dielectric and calorimetric α-relaxation maps for the tested systems is presented in Figure 5.

As can be observed in Figure 5, there is no decoupling between dielectric and calorimetric relaxation times, where the latter are in accord with the VFT extrapolations of the dielectric data to lower temperatures. Thus, calorimetric relaxation times imply the same dynamic fragility values as those established from dielectric measurements. This suggests that the molecular mobility reflected in structural relaxation does not govern the physical stability of the examined materials in the supercooled liquid state.

### 2.3. Thermodynamic Fragility (Calorimetric Study)

Since the dynamic fragility *m* as determined directly from dielectric measurements cannot predict the physical stability of the tested materials, it is interesting to check whether certain thermodynamic measures of this fragility, based on thermal quantities evaluated from calorimetric measurement, correlate with the tendency to crystallize. There have been a lot of efforts to define a thermodynamic measure of fragility and predict the parameter *m* using calorimetric methods, as well as to find a proper correlation between dynamic fragility and thermodynamic fragility [58,59,60]. An empirical relation between dynamic and thermodynamic fragility has been proposed by Wang and Angell [60,61]: $m=56T_{g}\Delta C_{P}/\Delta H_{m}$. They involved the heat capacity jump Δ*C*_p_ at the glass transition temperature, *T*_g_, as well as the fusion enthalpy, Δ*H*_m_. Based on the Random First-Order Transition (RFOT) theory, Lubchenko and Wolynes [62] derived a similar correlation, $m=34.7T_{m}\Delta C_{P}/\Delta H_{m}$, where *T*_m_ is the melting temperature. It is worth noting that the Lubchenko-Wolynes equation results from the Wang-Angell model assuming a widely used empirical relation *T*_m_ ≈ 2/3 *T*_g_. Both the thermodynamic measures of fragility have been tested by us, providing ambiguous predictions about the tendency to crystallize. Most likely, the measures failed due to the lack of contribution from some distribution of relaxation times of supercooled liquid during glass formation. Such a contribution is considered in another thermodynamic fragility expression that is explored in detail herein. 

Moynihan et al. [63,64] assumed that the width of the glass transition in the temperature dependencies of the heat capacity *C*_p_ obtained from calorimetric measurement could be reflected in the fragility parameter. This is due to some distribution of relaxation times of supercooled liquid during glass formation. A strong glass former exhibits a “lazy” change in structural relaxation times with temperature change, whereas fragile liquids show a rapid change in molecular mobility with temperature change. As a consequence, strong liquids reveal a wide glass transition, while fragile liquids have a much narrower glass transition [65]. Exploiting the relation between the dynamic fragility *m* and the apparent activation enthalpy for structural relaxation ΔH* at *T*_g_ gives the following:

(4) $$m=\frac{\Delta H^{*}\left(T_{g}\right)}{\ln(10)RT_{g}},$$

where ΔH*(*T_g_*) = *Rd*ln*τ*_α_/*d*(1/T) is defined [66] at a constant pressure and *R* is the gas constant. We evaluated fragility parameters for the examined materials by using an empirical relation [65,67] between ΔH*(*T*_g_) and the glass transition width in the temperature dependence of *C*_p_,

(5) $$\Delta H^{*}\left(T_{g}\right)=RC\frac{T_{g}^{onset}T_{g}^{end}}{T_{g}^{end}-T_{g}^{onset}},$$

where $T_{g}^{onset}$ and $T_{g}^{end}$ denote, respectively, temperatures of the onset and end of the glass transition in DSC thermogram, while the constant *C* = 5 [65].

**Figure 6:**
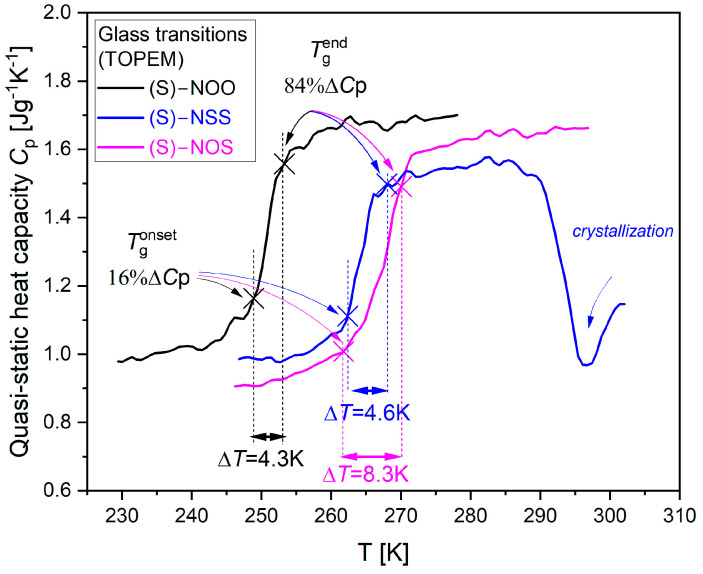
Temperature dependencies of quasi-static specific heat capacity *C*_p_ for the examined materials near their glass transitions, evaluated from stochastic temperature-modulated differential scanning calorimetry (TOPEM^®^). The illustration of the method estimation of the glass transition width, ∆*T*, is the temperature interval within which the heat capacity *C*_p_ changes from 16% to 84% of the total heat capacity step ∆*C*_p_ at *T*_g_ (taken from the Donth model) [68,69].

In order to accurately determine the thermodynamic fragility based on Equation (4), we evaluated the temperature dependencies of quasi-static specific heat capacity for the examined materials near their glass transitions using stochastic temperature-modulated differential scanning calorimetry (TOPEM^®^). In our experiments, the quenched samples were heated at a rate of 0.5 K/min. We adjusted our evaluations using a sapphire reference curve. The experimental dependencies of quasi-static specific heat capacity are presented in Figure 6 for each investigated compound. 

As can be observed, the temperature dependencies of the quasi-static heat capacity *C*_p_ show sigmoidal changes for each compound, which reflects liquid-glass transitions. The values of the glass transition temperatures *T*_g_ (evaluated as the temperature of the half-step height of the quasi-static heat capacity *C*_p_) and heat capacity steps Δ*C*_p_ at *T*_g_ for all examined materials are collected in Table 3.

To evaluate thermodynamic fragility using the Moynihan model (Equations (4) and (5)), the width of the glass transition needs to be analyzed. Comparing the obtained dependencies (*C*_p_(*T*)), one can observe that the width of the glass transition is much larger for (S)-NOS, which does not reveal any tendency to crystallize, than those for the easily crystallizing compounds, (S)-NOO and (S)-NSS. We assumed the glass transition width ∆*T* as the temperature interval within which the heat capacity *C*_p_ changes from 16% to 84% of the total heat capacity step ∆*C*_p_ at *T*_g_. This method was taken from the Donth model, which is often used to evaluate the numbers of dynamically correlated molecules based on the width of the glass-liquid transition [68,69]. The illustration of the estimation of the glass transition width ∆*T* is presented in Figure 6. Onset of the glass transition $T_{g}^{onset}$ is the temperature at which *C*_p_ = 16%∆*C*_p_, whereas the end of the glass transition $T_{g}^{end}$ is the temperature at which *C*_p_ = 84%∆*C*_p_. We found that the width of the glass transition is the largest ∆*T* = 8.3 K for the physically stable compound (S)-NOS, while for compounds with a high tendency to crystallize, such as (S)-NSS and (S)-NOO, the width of the glass transition is significantly smaller and equal to ∆*T* = 4.6 K and ∆*T* = 4.3 K, respectively. The established values of $T_{g}^{onset}$ and $T_{g}^{end}$, collected in Table 3, were then used to determine the activation enthalpy for structural relaxation Δ*H* at *T*_g_, which for materials with high physical stability was revealed to be almost half that of crystallizing ones. 

**Figure 7:**
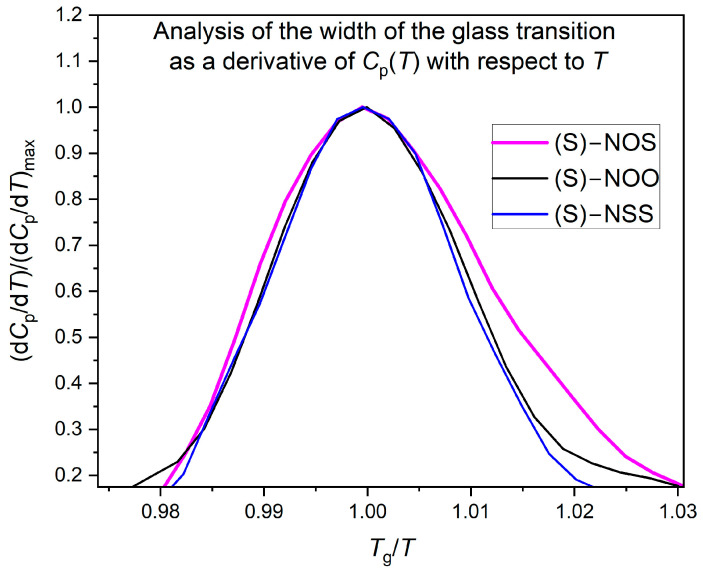
Analysis of the glass transition width of the tested materials based on the first derivative of their temperature dependences of specific heat: the diagram shows the first derivative of *C*_p_(*T*) with respect to temperature scaled by maximal values of d*C*_p_/d*T* as a function of scaled temperature *T*_g_/*T*.

It is worth noting that the assessment of the width of the glass transition does not depend on the method of determining the $T_{g}^{onset}$ and $T_{g}^{end}$ values. Analyzing the graph of the derivative of specific heat vs. the scaled temperature *T*_g_/*T* (see Figure 7), it is visible that the width of the bell curve for the physically stable compound (S)-NOS is larger than those for the crystallizing substances (S)-NOO and (S)-NSS.

The tested substance (S)-NOS with high physical stability (CLASS III) is characterized by a low thermodynamic fragility value (*m* = 69), while almost twice as high values (*m* = 125) were obtained for materials with a high tendency to crystallize (CLASS I and CLASS II). The obtained fragility tendency from the Moynihan model (see Table 2), based on the width of the glass transition, correlates with the standard viewpoint that considers the fragility parameter as an accurate predictor of physical stability, as well as Tanaka’s concept of frustration against crystallization [36,37]. According to this two-order-parameter (TOP) model, which is a simulation-based attempt to explain the capability, a supercooled liquid near the glass transition tends to order into the equilibrium crystal (long-range ordering). However, the liquid does not undergo crystallization due to frustration, characterized by locally favored short-range ordering. This frustration creates a higher free-energy barrier for nucleation and hinders crystallization by acting as an impurity. Fragile liquids exhibit weaker frustration against crystallization. Consequently, a fragile system is more prone to crystallizing than a strong glass-former.

### 2.4. Isothermal Crystallization Kinetics (Dielectric Study)

**Figure 8:**
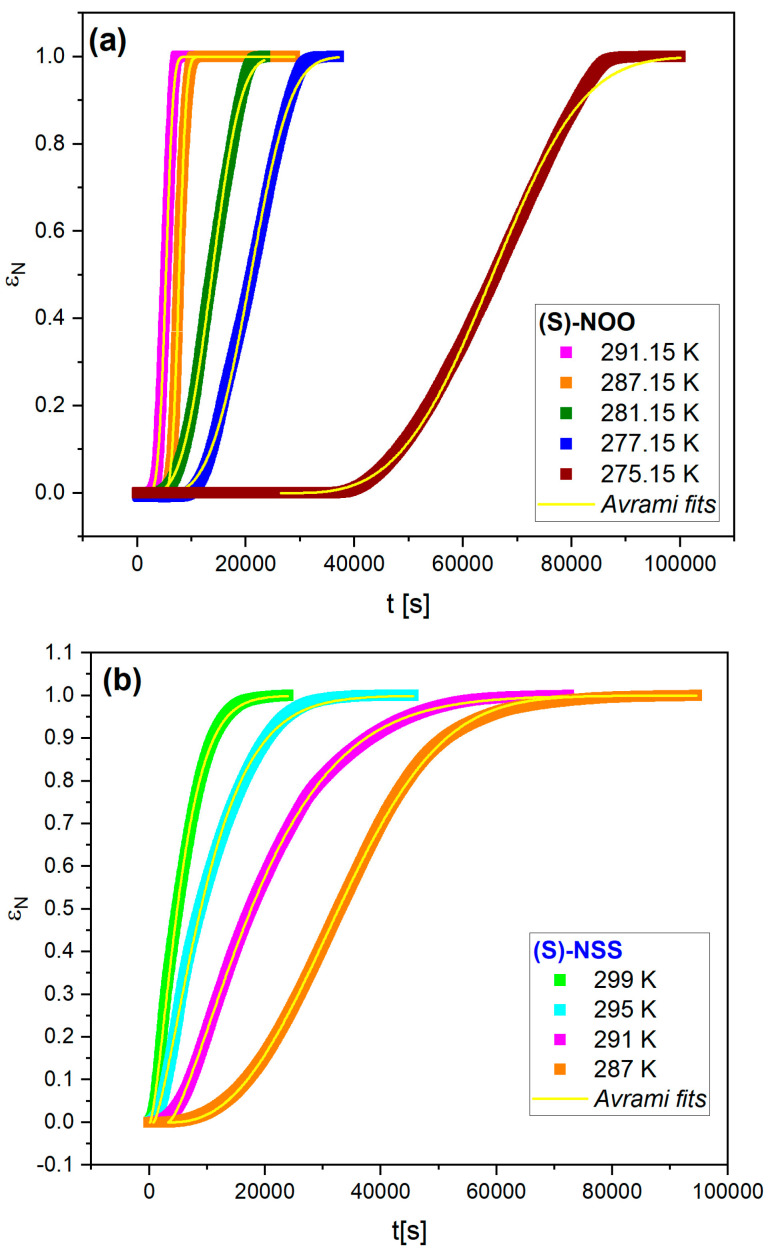
Time dependencies of normalized static permittivity ε_N_(t) for (S)-NOO (**a**), and (S)-NSS (**b**). Solid lines represent Avrami fits in terms of Equation (7), with its parameters collected in Table 4.

As well as the tendency to crystallize, we also investigated isothermal crystallization kinetics using broadband dielectric spectroscopy. The molecular mobility is gradually reduced during the crystallization process. It can be monitored by the BDS technique as a decrease in dielectric strength (∆*ε* = *ε*_s_ − *ε*_∞_, where *ε*_s_ and *ε*_∞_ are values of the real part of the dielectric permittivity *ε*’ in the limit of low and high frequency, respectively). The isothermal crystallization of (S)-NOO and (S)-NSS have been investigated at several temperatures above *T*_g_ at constant frequencies as a function of time. Isothermal crystallization of (S)-NOS has not been observed in a relatively short time to be analyzed. Before crystallization, each sample was vitrified on the capacitor plate. Immediately after vitrification, the static dielectric permittivity *ε*_s_(*t*) was recorded under isothermal conditions at a constant frequency and at specified time intervals throughout the crystallization process. Isothermal crystallization kinetics curves (showing the increase in the crystallization degree with time) were obtained via normalization of experimental data using the following formula [70]:

(6) $$\varepsilon_{N}\left(t\right)=\frac{\varepsilon_{s}\left(0\right)-\varepsilon_{s}\left(t\right)}{\varepsilon_{s}\left(0\right)-\varepsilon_{s}\left(\infty \right)},$$

where *ε*_s_(0) is the static dielectric permittivity at the beginning of the crystallization, *ε*_s_(∞) is the corresponding value after the end of the crystallization process, and *ε*_s_(*t*) is the static permittivity at a given time *t* of crystallization. Figure 8a,b present the plots of normalized static permittivity *ε*_N_(*t*) as a function of time at a given crystallization temperature for (S)-NOO and (S)-NSS, respectively.

**Figure 9:**
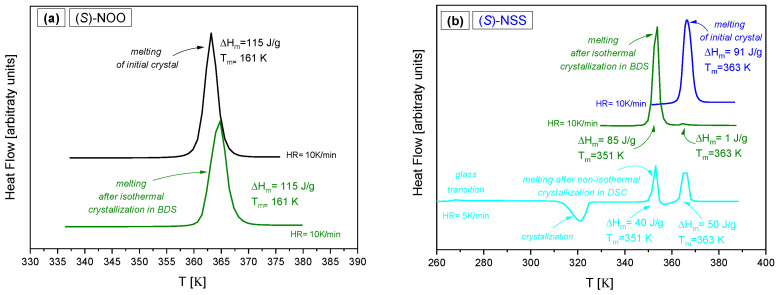
Comparison of DSC thermograms of the melting processes of initial crystals and after cold crystallization for (**a**) (S)-NOO, and (**b**) (S)-NSS.

It is worth emphasizing that after measuring the isothermal crystallization kinetics at each temperature, the degree of crystallization and the crystalline form to which the sample transformed from the supercooled liquid state was checked. For both (S)-NOO and (S)-NSS, we confirmed crystallization in the entire sample volume (with an accuracy of 5%). It turned out that after isothermal crystallization kinetics in a supercooled liquid, (S)-NOO returns to the initial crystal form with the same melting temperature and heat of fusion (see Figure 9a), while (S)-NSS is transformed into a more complex polymorphic form (see Figure 9b), consisting of at least two crystalline forms differing in melting temperature. One of them is similar to the initial crystal; it has also been observed under non-isothermal crystallization in the DSC experiment. 

The global kinetics of isothermal crystallization have been analyzed using the Avrami model [71,72], which provides important information about the crystallization mechanisms. According to this model, the normalized static permittivity changes with time are as follows:

(7) $$\varepsilon_{N}\left(t\right)=1-exp\left(-K{\left(t-t_{0}\right)}^{n}\right),$$

where *K* = *k*^n^ and *k* is a crystallization rate constant, which depends on the crystallization temperature and geometry of the sample, *n* is the Avrami exponent that is related to the time dependence of the nucleation rate and the dimensionality of the crystallization, and *t*_0_ is the induction time of crystallization, defined as the time from the beginning to the point at which a stable crystal nucleus starts growing. The values of Avrami parameters, *n* and *k*, collected in Table 4, were used to describe the entire time dependencies of *ε*_N_(*t*) (see solid lines in Figure 8a,b).

**Figure 10:**
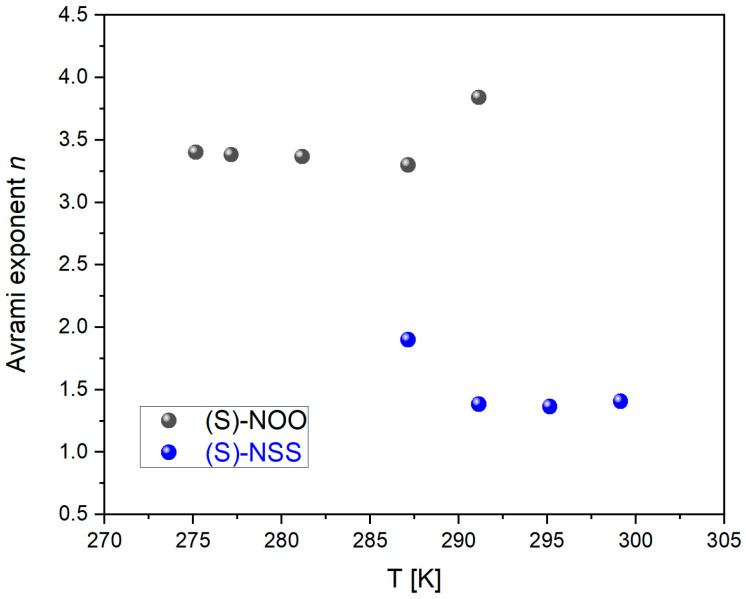
Dependencies of the Avrami exponent *n* on temperature T for (S)-NOO and (S)-NSS.

Analyzing the obtained values of the Avrami equation parameters, we start with the values of the Avrami exponent *n*, which considerably differs between the examined materials. For (S)-NOO, the values of *n* vary between 3.40 and 3.84, whereas in the case of (S)-NSS, they are lower and range between 1.36 and 1.90, as can be seen in Table 4 and Figure 10. Such results suggest that (S)-NOO can form co-existent three-dimensional crystallites, whereas (S)-NSS transforms to mainly one-dimensional crystallites, with instantaneous and sporadic nucleation most likely occurring in both materials [73,74,75,76].

**Figure 11:**
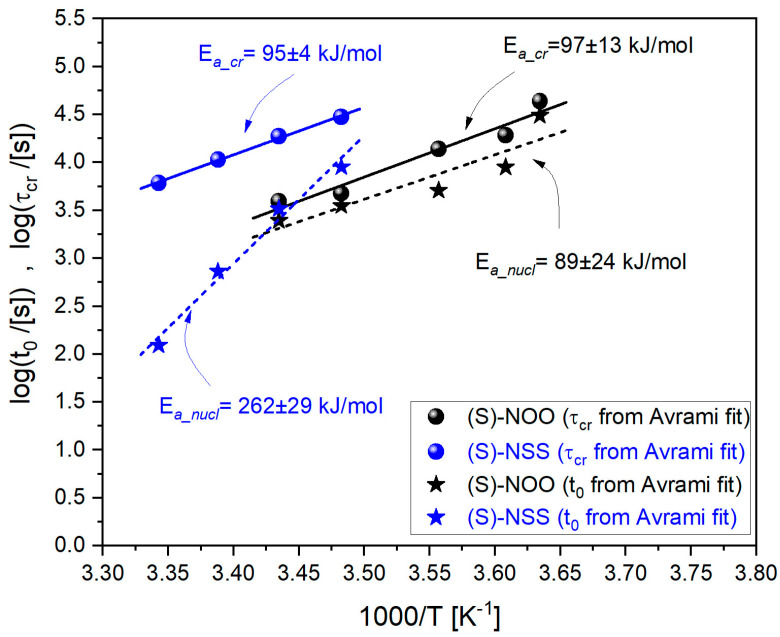
Dependencies of logarithms of the induction time *t*_0_ and the crystallization time *τ*_cr_ on the inverse temperature for (S)-NOO and (S)-NSS.

To investigate the crystallization timescales based on the Avrami model, it is convenient to consider the crystallization time *τ*_cr_ = 1/*k* instead of the crystallization rate *k*, which enables us to compare the timescales of the induction of crystallization and the overall crystallization. In most experimental cases, both the induction time t_0_ and the crystallization time *τ*_cr_ obey the following Arrhenius law:

(8) $$\log y=logy_{\infty }+\frac{E_{a\_x}}{RT}\log e$$

where *y* is the induction time *t*_0_ or the crystallization time *τ*_cr_ = 1/*k*, *y*_∞_ is the timescale in the limit of high temperatures, *E_a_x_* is the activation energy for nucleation or overall crystallization, and the postfix *x* represents *cr* or *nucl*, respectively. As can be seen in Figure 11, one can assume that the parameters *t*_0_ and *τ*_cr_ obtained for (S)-NOO and (S)-NSS comply with Equation (8) to a sufficiently good approximation, including *t*_0_ for (S)-NSS, despite some deviations from the linear dependence. 

Similarly to the tendency to crystallize, the crystallization timescales of (S)-NOO and (S)-NSS reveal some differences. Interestingly, for (S)-NOO, the induction time *t*_0_ is slightly less than the crystallization time *τ*_cr_. However, the nucleation and overall crystallization activation energies are relatively small and close to each other for this material (*E*_a_nucl_ = 89 ± 24 and *E*_a_cr_ = 97 ± 13). This means that the long induction time does not significantly affect the energetically favorable overall crystallization process. On the other hand, the activation energy for nucleation of (S)-NSS, *E*_a_nucl_ = 262 ± 29, is almost three times higher than that for (S)-NOO, which shows that the nucleation of (S)-NSS is more difficult compared to that of (S)-NOO. It is worth noting that this is accompanied by the lower value of the Avrami parameter *n* of (S)-NSS as compared to that of (S)-NOO, which reflects, among other things, the different dimensionality of crystal growth of the materials (one-dimensional and three-dimensional ones, in (S)-NSS and (S)-NOO, respectively). Such differences in the nucleation barriers can precede the formation of variant crystalline structures. Nevertheless, the activation energies for overall crystallization of (S)-NSS and (S)-NOO are almost identical: *E*_a_cr_ = 95 ± 4 and *E*_a_cr_ = 97 ± 13, respectively. Such a small value of *E*_a_cr_ for (S)-NSS compared to the relatively large value of *E*_a_nucl_ indicates that the crystal growth may occur quickly after forming crystal nuclei. 

In addition to structural relaxation (discussed in the previous sections), molecular mobilities reflected in secondary relaxations are considered to influence the crystallization process. The origin of secondary relaxations observed in the examined materials has been classified as intramolecular. In general, intramolecular motions could affect the crystallization process. However, the values obtained for the nucleation and overall crystallization activation energies are considerably higher than those for the activation energies of secondary relaxations. Therefore, the relation between secondary relaxation observed in the examined materials and the crystallization process is ambiguous. 

## 3. Materials and Methods

### 3.1. Materials

The chemical structures of the examined materials that were purchased from the Merck Group, Germany, Darmstadt, in crystalline form are presented in Table 2. They are organic compounds with some common structural features: (i) All three compounds share the common feature of containing a benzyl group, which is a phenyl group (-C_6_H_5_) attached to a methylene group (-CH_2_). (ii) All substances contain a heterocyclic ring in their structure. Specifically, they have either an oxazolidine or thiazolidine ring. These rings consist of carbon, nitrogen, and either oxygen (in oxazolidines) or sulfur (in thiazolidines) atoms. (iii) They are all chiral compounds with an “(*S*)” configuration, signifying a specific spatial orientation of substituents around a chiral center within their respective ring structures. They have a non-superimposable mirror image, which is a characteristic feature of chiral compounds. The purities of the examined materials, (S)-4-Benzyloxazolidine-2-thione (S-NOS), (S)-4-Benzyl-2-oxazolidinone (S-NOO), and (S)-4-Benzylthiazolidine-2-thione (S-NSS), are as follows: ≥97%, =99%, and ≥95%, respectively. 

### 3.2. Standard Differential Scanning Calorimetry

Calorimetric measurements of the investigated materials were carried out using a Mettler-Toledo DSC apparatus equipped with a liquid nitrogen cooling accessory and an HSS8 ceramic sensor (heat flux sensor with 120 thermocouples). Temperature and enthalpy calibrations were performed using indium and zinc standards. The amorphous systems were prepared via quench-cooling of the supercooled liquid phase. Each sample was prepared in a measuring aluminum crucible (40 μL). Crucibles with samples were sealed at the top, with one puncture. 

Standard DSC measurements were used to determine thermodynamic quantities characterizing the crystalline phase, as well as the crystallization tendency of each supercooled material under non-isothermal conditions. For this purpose, each initial crystalline sample was first melted and then subjected to multiple cooling and heating cycles (below glass transition temperatures and above melting points) at different rates (from three to 50 K/min). For a single cycle, the cooling rate (CR) was always equal to the heating rate (HR). Each measurement at a given cooling/heating rate was repeated three times to confirm repeatability.

### 3.3. Stochastic Temperature-Modulated Differential Scanning Calorimetry (TOPEM^®^)

To determine the accurate temperature dependencies of the isobaric heat capacity of examined compounds both in the crystalline and glassy states near their glass transitions, we exploited the stochastic temperature-modulated differential scanning calorimetry (TMDSC) technique implemented by Mettler-Toledo (TOPEM^®^). The crystalline and quenched samples were heated at a rate of 0.5 K/min. In the experiment, a temperature amplitude of the pulses of 0.5 K was selected, with a switching time range with minimum and maximum values of 15 and 30 s, respectively. We adjusted our evaluations of the temperature dependence of the quasi-static heat capacity *C*_p_(*T*) using a sapphire reference curve. Each measurement was repeated three times to confirm repeatability.

To evaluate the calorimetric structural relaxation times of investigated materials near the glass transition, we used isobaric heat capacity calibrated with sapphire. We established that the temperature dependencies of the real part of the complex heat capacity *C*′_p_(*T*) are frequency-dependent within the temperature window of each sigmoidal change in the heat capacity for all investigated materials. It was also observed that the sigmoidal changes in the dependencies *C*′_p_(*T*) shift towards high temperatures with increasing frequency. This behavior is characteristic of relaxation processes. The calorimetric structural relaxation times *τ*_α_ = 1/2π*f* were determined from the temperature dependencies of the real part of the complex heat capacity *C*′_p_(*T*), obtained at different frequencies in the glass transition region. The glass transition temperature *T*_g_ was determined for each frequency as the temperature of the half-step height of *C*′_p_(*T*).

### 3.4. Broadband Dielectric Spectroscopy

Isobaric measurements of the dielectric permittivity ε*(*f*) = ε′(*f*) – *i*ε″(*f*) were performed using the Novo-Control Alpha dielectric spectrometer over the frequency range (10^−2^–10^6^) Hz and in the wide temperature range at ambient pressure. Non-isothermal dielectric measurements of examined materials were performed in a parallel-plate cell immediately after melting its crystalline form. The sample temperatures were controlled by Quatro System using a nitrogen gas cryostat. The temperature stability was better than 0.1 K. 

## 4. Conclusions

Investigations of the physical stability of tested low molecular weight organic materials with a similar chemical structure have shown that their tendency to crystallize is completely different and can be classified into three different crystallization classes:

(i) For (*S*)-4-Benzyl-2-oxazolidinone [(S)-NOO)], the crystallization process is observed during cooling from the melt;
(ii) (*S*)-4-Benzylthiazolidine-2-thione [(S)-NSS] does not crystallize during cooling from the melt to below *T_g_*, but easily crystallizes during subsequent reheating above *T*_g_;
(iii) For (*S*)-4-Benzyloxazolidine-2-thione ((S)-NOO), no crystallization is observed, either during the quenching from the melt or during the reheating cycle.

The results obtained from the study of isothermal crystallization kinetics have followed the tendency to crystallize, which is especially observed in the nucleation and overall crystallization activation energies. The former for (S)-NSS is about three times larger (*E*_a_nucl_ = 262 ± 29) than *E*_a_nucl_ = 89 ± 24 for (S)-NOO. The differences in *E*_a_nucl_ are accompanied by variant dimensionality in crystal growth, which is one-dimensional in (S)-NSS and three-dimensional in (S)-NOO, as inferred from the values of the Avrami parameter *n*. Such disparate nucleation barriers can reflect the formation of different crystalline structures in the materials. However, the overall crystallization energy is small and almost the same for both the crystallizing materials: *E*_a_cr_ = 95 ± 4 and *E*_a_cr_ = 97 ± 13, for (S)-NSS and (S)-NOO, respectively. This suggests that the overall crystallization process in these materials is energetically favorable despite the differences in the nucleation barriers. 

Based on dielectric measurements, it was shown that all tested substances are characterized by the same dynamic fragility (*m* = 93). The result has been confirmed by the Angell plot, in which the dependencies of relaxation times as a function of *T*_g_/*T* superimpose for all the tested compounds. Calorimetric relaxation times imply the same dynamic fragility values as those established from dielectric measurements. This is because extrapolations of the dielectric α-relaxation times to lower temperatures match the temperature dependencies of the calorimetric α-relaxation times for studied materials. The identical values of the dynamic fragility parameters do not correlate with the completely different crystallization tendencies of the examined materials. While dynamic fragility is not related to the tendency to crystallize, in the case of thermodynamic fragility, a certain correlation can be established. 

From the thermodynamic fragility measures we analyzed, we noticed that the thermodynamic fragility from the Moynihan model, based on the width of the glass transition in the temperature dependences of *C*_p_, correlates best with the tendency to crystallize. We obtained a relatively small value of fragility (*m* = 69) for the compound that does not tend to crystallize, (S)-NOS, and much higher values (*m* = 125) for the substances that readily crystallize, (S)-NOO and (S)-NSS. A small value of the *m* parameter is associated with a wide glass transition. The thermodynamic fragility of *m* = 125 for (S)-NOO and (S)-NSS allows us to classify this drug as a fragile liquid. The large value of *m* indicates a large average degree of molecular mobility of structural relaxation near the glass transition and correlates with the large tendency of these materials to crystallize. This result correlates also with the two-order-parameter (TOP) model. Some frustrations caused by a locally favored short-range ordering of fragile systems against crystallization are weaker than those of strong materials. Therefore, fragile systems should have a weaker glass-forming ability and easier crystallization than strong glass formers.

The obtained results should be a motivation to conduct additional analyses of thermodynamic fragility related to the width of the glass transition for a larger number of compounds with different crystallization tendencies to verify whether this factor can correctly predict the physical stability of supercooled liquids and glasses.

## Figures and Tables

**Table 1:** Values of the VFT parameters were found by fitting the experimental dependencies presented in Figure 3 to Equation (3), as well as glass transition temperatures *T*_g_ from BDS data analysis for *τ*_α_ = 100 s.

Material	Physical Stability	VFT Parameters			*T*_g_ [K] BDS (for *τ*_α_ = 100 s)
		log(*τ*_0_/s)	*A* [K]	*T*_0_ [K]	
(S)-NOS	Class III	−14.77 ± 0.24	1860 ± 80	218 ± 2	265.9 ± 0.2
(S)-NSS	Class II	−14.80 ± 0.02	1830 ± 30	216 ± 1	262.8 ± 0.1
(S)-NOO	Class I	−14.70 ± 0.01	1720 ± 20	204 ± 1	248.9 ± 0.1

**Table 2:** Comparison of the dynamic fragility parameters m for *τ*_α_ = 100 s, strength parameters *D* from the VFT equation, values of activation energy of structural relaxation *E_a_* at *T_g_* for examined materials, and the thermodynamic fragility.

Material	Molecular Weight [g/mol] & Chemical Structure	Physical Stability	*E*_a_ at *T*_g_ [kJ/mol] (for *τ*_α_ = 100 s)	Strength Parameter *D*	Dynamic Fragility *m* (for *τ*_α_ = 100 s)	Thermodynamic Fragility Moynihan Model
(S)-NOS	Mw = 193.27 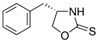	Class III	471 ± 8	8.55 ± 0.43	93 ± 2	69 ± 2
(S)-NSS	Mw = 209.33 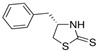	Class II	470.0 ± 0.7	8.48 ± 0.25	93 ± 1	125 ± 4
(S)-NOO	Mw = 177.20 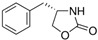	Class I	443.4 ± 0.3	8.40 ± 0.13	93 ± 1	125 ± 4

**Table 3:** Quantities obtained from calorimetric measurements (standard DSC and TOPEM^®^), needed to calculate the analyzed thermodynamic fragility.

Material	*T*_m_ [K] Standard DSC	Δ*H*_m_ [J/g] Standard DSC	*T*_g_ [K] TOPEM^®^	Δ*C*_p_ [J/gK] TOPEM^®^	$T_{g}^{onset}$ [K]	$T_{g}^{end}$ [K]	ΔH* at *T*_g_ ^(a)^ [kJ/mol]
(S)-NOS CLASS III	338.7 ± 0.5	61 ± 1	268.0 ± 0.1	0.61 ± 0.03	261.8 ± 0.1	270.1 ± 0.1	354.6
(S)-NSS CLASS II	363.8 ± 0.5	91 ± 2	263.9 ± 0.1	0.57 ± 0.03	263.4 ± 0.1	268.0 ± 0.1	635.1
(S)-NOO CLASS I	361.1 ± 0.5	115 ± 3	251.0 ± 0.1	0.58 ± 0.03	248.9 ± 0.1	253.2 ± 0.1	602.4

^(a)^ → The evaluation accuracy of ΔH* at *T*_g_ has not been shown because it has not been straightforwardly used to determine the evaluation accuracy of the thermodynamic fragility, which is calculated from Equation (4) after incorporating Equation (5).

**Table 4:** Comparison of isothermal crystallization kinetics parameters for (S)-NOO and (S)-NSS, obtained from the Avrami analysis.

	(S)-NOO		
**Crystallization Temperature** **[*K*]**	***n***	***t*_0_** **[*s*]**	***k*** **[*s*^−1^]**
291.15	3.84 ± 0.11	2458 ± 99	2.54·10^−4^ ± 6.6 × 10^−6^
287.15	3.30 ± 0.13	3533 ± 15	2.11·10^−4^ ± 1.5 × 10^−6^
281.15	3.37 ± 0.06	5100 ± 220	7.26·10^−5^ ± 1.2 × 10^−6^
277.15	3.38 ± 0.07	8900 ± 330	5.22·10^−5^ ± 9.2 × 10^−7^
275.15	3.40 ± 0.15	30,900 ± 100	2.29·10^−5^ ± 5.6 × 10^−8^
	(S)-NSS		
299.15	1.41 ± 0.01	124 ± 25	1.64·10^−4^ ± 9 × 10^−7^
295.15	1.36 ± 0.01	727 ± 40	9.38·10^−5^ ± 5 × 10^−7^
291.15	1.38 ± 0.01	3284 ± 24	5.36·10^−5^ ± 9 × 10^−8^
287.15	1.90 ± 0.05	8959 ± 67	3.36·10^−5^ ± 9 × 10^−8^

## Data Availability

The data presented in this study are available from the corresponding author upon request.
